# Supplementary material for: pH-Responsive Hybrid Nanoassemblies for Cancer Treatment: Formulation Development, Optimization, and In Vitro Therapeutic Performance
Source: Pharmaceutics. 2023 Jan 18;15(2):326. doi: 10.3390/pharmaceutics15020326 (PMC9966415; doi:10.3390/pharmaceutics15020326)
Supplement: Supplementary file 1 [file pharmaceutics-15-00326-s001.zip › pharmaceutics-2142577-supplementary.pdf]

pharmaceutics

MDPI

Supplementary Materials: pH-Responsive Hybrid Nanoassemblies for Cancer Treatment: Formulation Development, Optimization, and In Vitro Therapeutic Performance

Patrícia V. Teixeira [1,2], Filomena Adega [3,4], Paula Martins-Lopes [2,4], Raul Machado [5,6], Carla M. Lopes [7,*] and Marlene Lúcio [1,5,*]

Table S1. Fittings of DOX release kinetics of cubosomes in buffer with pH of 5.5 and 7.4 using mathematical models.

1st Order Model	pH	Parameters	R^2	R^2 Adjusted
$Fmax(1 - e^{-kt})$	5.5	$Fmax = 23.3 \pm 2.4$<br>$k = 0.63 \pm 0.24$	0.937	0.932
	7.4	$Fmax = 8.05 \pm 0.8$<br>$k = 0.88 \pm 0.33$	0.912	0.906
**Weibull model**				
$Fmax(1 - e^{(-c_2 t^{c_3})})$	5.5	$Fmax = 24.5 \pm 2.7$<br>$c_2 = 0.60 \pm 0.17$<br>$c_3 = 0.76 \pm 0.24$	0.950	0.942
	7.4	$Fmax = 8.5 \pm 0.9$<br>$c_2 = 0.78 \pm 0.22$<br>$c_3 = 0.70 \pm 0.25$	0.935	0.924
**Gallagher - Corrigan model**				
$Fmax(1 - e^{-k_1 x}) + (Fmax - Fb)(\frac{e^{-k_2 x - k_2 Tmax}}{1 + e^{-k_2 x - k_2 Tmax}})$	5.5	$Fmax = \sim -239.0$<br>$Fb = 23.3$<br>$Tmax = \sim -212.6$<br>$k1 = 0.63$<br>$k2 = \sim -4.94$	0.9370	0.9118
	7.4	$Fmax = \sim -2413709209$<br>$Fb = 8.039$<br>$Tmax = \sim -6587534350$<br>$k1 = 0.8813$<br>$k2 = \sim -124612968$	0.9124	0.87736

$\boldsymbol{k}$ is the first-order release constant; $\boldsymbol{Fb}$ is the amount of drug released directly from the surface of the nanosystem (during the initial burst); $\boldsymbol{Fmax}$ is the amount of drug released during the process; T_{max} is the time in which the maximum release of the drug from the surface of the nanosystem occurs (after the initial burst) and $\boldsymbol{k_1}$ and $\boldsymbol{k_2}$ are release constants of first and second phase, respectively.

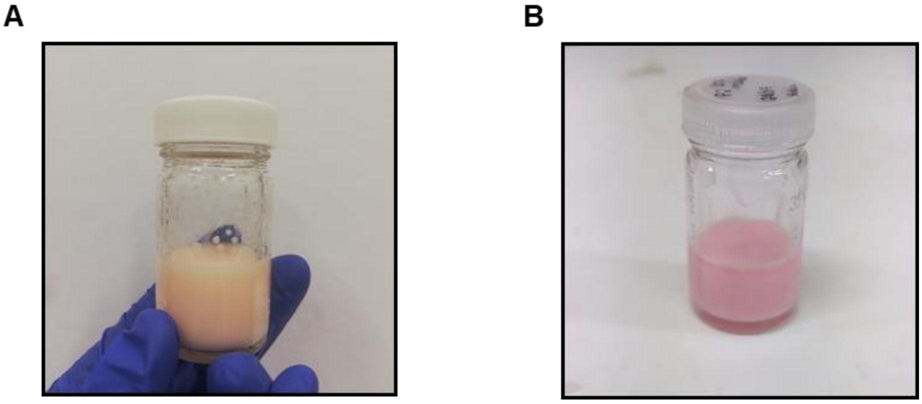

Figure S1: Macroscopic changes in DOX-LNLCs: M3 by direct-mixing loading method over 4 weeks. (**A**) After preparation of formulation; (**B**) After 4 weeks storage at 25°C.

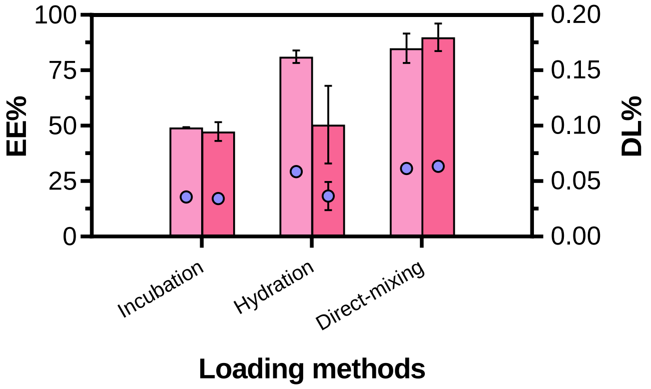

Figure S2: Encapsulation efficiency (EE%) and drug loading (DL%) of DOX-LNLCs by different loading methods. Pink data represents the DOX-LNLCs (LNLCs prepared by M2 are lighter pink, while M3 is darker). The data were expressed as mean ± standard deviation of three independent measurements.

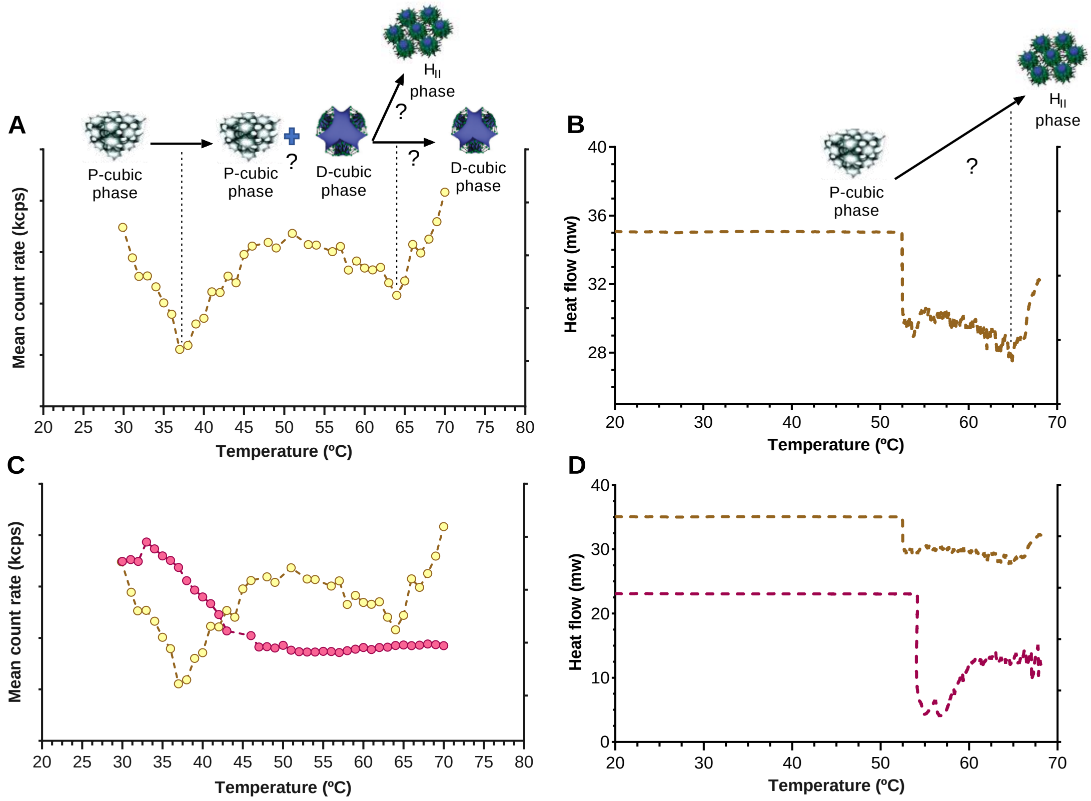

Figure S3: Thermodynamic study of phase transition temperature of the empty-LNLCs: M3 (yellow data) or the DOX-LNLCs: M3 (pink data) obtained by DLS method by the measurement of mean count rate as a function of temperature (**A** and **C**) or by DSC by the measurement of heat flow into or out of a sample as a function of temperature (**B** and **D**).

The DLS thermodynamic characterization of empty-LNLCs: M3 showed a first transition between 35 and 40 °C and a second transition phase beginning at 50 °C and occurring at about 62 °C (Figure S3A). According to the X-ray diffraction studies performed by Wörle et al., the first transition at 35–40 °C may be between a cubic P-type phase to a coexistence of a P- and D-type phase, whereas the second transition may be a transition to a D-type phase [67]. Another possibility reported by other authors in LNLCs with 20% (w/w) of P407 is that the cubic crystal is gradually transformed into a hexagonal liquid crystal when the temperature exceeded 60 °C [68]. Therefore, the second phase transition detected in the DLS measurements for empty-LNLCs: M3 may also be attributed to this transition. In fact, DSC measurements (Figure S3B) corroborate this second assumption. Unlike the GMO, which has no clear endothermic peak, the thermogram of P407 (data not shown) has an endothermic peak at 56 °C [69]. It is worth noting that the phase transitions between different types of cubic phases at 35–40 °C observed by DLS and reported by X-ray diffraction studies [67] were not distinguishable in the DSC measurements performed herein and in none of the reported DSC measurements [49,68–70]. This is due to the different sensibilities of the techniques used, as DSC only detects major transitions, whereas X-ray diffraction studies and DLS can detect subtle changes.

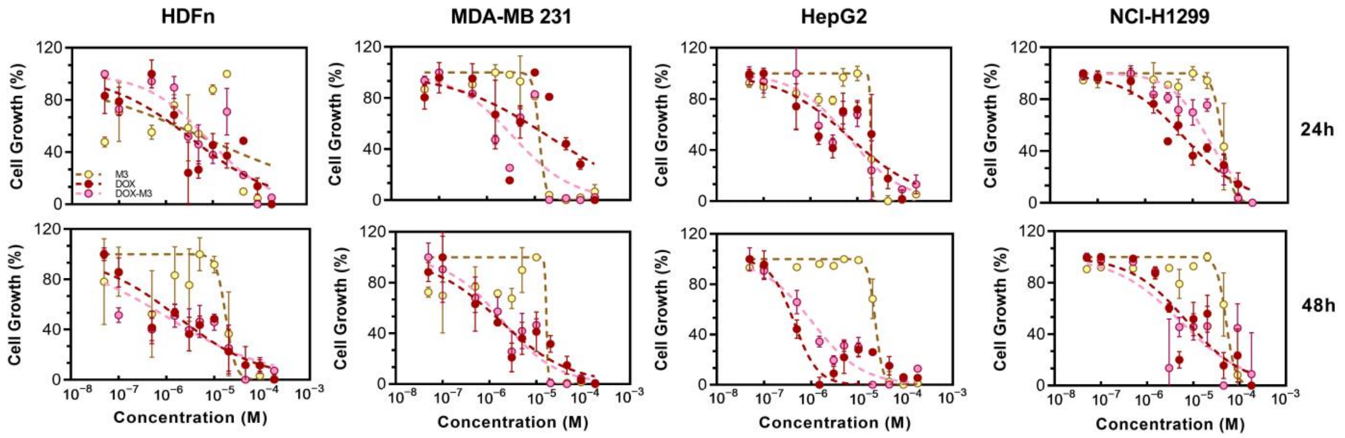

Figure S4: Evaluation of in vitro cytotoxicity of LNLCs: M3 containing DOX (0.98 mg/mL) on HDFn, MDA-MB-231, HepG2 and NCI-H1299 cell lines as a function of DOX concentration and incubation time (24 and 48 hours). Yellow data are the empty-LNLCs: M3 obtained by the emulsification method; pink data represents the DOX-LNLCs; and red data are the free DOX (0.98 mg/mL). The data were expressed as mean ± standard deviation of three independent experiments.

References

49. Jin, X.; Zhang, Z.-h.; Li, S.-l.; Sun, E.; Tan, X.-b.; Song, J.; Jia, X.-b. A nanostructured liquid crystalline formulation of 20(S)-protopanaxadiol with improved oral absorption. *Fitoterapia* **2013**, *84*, 64-71, doi:10.1016/j.fitote.2012.09.013.

67. Wörle, G.; Drechsler, M.; Koch, M.H.J.; Siekmann, B.; Westesen, K.; Bunjes, H. Influence of composition and preparation parameters on the properties of aqueous monoolein dispersions. *International Journal of Pharmaceutics* **2007**, *329*, 150-157, doi:10.1016/j.ijpharm.2006.08.023.

68. Tian, Y.; Li, J.C.; Zhu, J.X.; Zhu, N.; Zhang, H.M.; Liang, L.; Sun, L. Folic Acid-Targeted Etoposide Cubosomes for Theranostic Application of Cancer Cell Imaging and Therapy. *Med Sci Monit* **2017**, *23*, 2426-2435, doi:10.12659/msm.904683.

69. Bei, D.; Zhang, T.; Murowchick, J.B.; Youan, B.-B.C. Formulation of Dacarbazine-loaded Cubosomes. Part III. Physicochemical Characterization. *AAPS PharmSciTech* **2010**, *11*, 1243-1249, doi:10.1208/s12249-010-9496-7.

70. Rarokar, N.R.; Saoji, S.D.; Raut, N.A.; Taksande, J.B.; Khedekar, P.B.; Dave, V.S. Nanostructured Cubosomes in a Thermoresponsive Depot System: An Alternative Approach for the Controlled Delivery of Docetaxel. *AAPS PharmSciTech* **2016**, *17*, 436-445, doi:10.1208/s12249-015-0369-y.
